# Probiotic-fermented herbal residues in obesity management: a review

**DOI:** 10.3389/fpubh.2026.1797885

**Published:** 2026-06-23

**Authors:** Xuejiao Tian, Zhigang An, Zhen Yang, Lin Xi, Liyue Yu, Yonge Gu, Xuefei Yang, Hongru Qin, Min Zhang, Jianjun Wu

**Affiliations:** School of Public Health, Gansu University of Traditional Chinese Medicine, Lanzhou, Gansu, China

**Keywords:** anti-obesity, Chinese herbal medicine residue, fermentation, healthcare, probiotics

## Abstract

The global prevalence of has reached epidemic proportions, largely driven by dietary shifts toward high-calorie, processed foods, and sedentary lifestyles. Obesity is a complex polygenic disorder characterized by excessive adipose tissue accumulation and adipocyte hypertrophy, leading to various metabolic dysfunctions. The gut microbiota plays a pivotal role in regulating host energy metabolism, and dysbiosis, an imbalance in its composition and function, is strongly linked to obesity development and progression, Traditional Chinese medicine (TCM) has long been utilized for weight management, yet “efficiency limitations” and “resource waste” remain significant concerns. This comprehensive review explores the emerging approach of using probiotic-fermented herbal residues for obesity management. We examine how fermentation technologies transform herbal byproducts into high-value anti-obesity preparations through biological processes that enhance bioactive compounds, improve bioavailability, and modulate the gut-liver axis. The integration of herbal medicine with modern biotechnology impossible represents a promising frontier in sustainable healthcare and precision medicine for metabolic disorders.

## 1 Introduction

An energy imbalance between food intake and energy expenditure can lead to obesity (1). Obesity is a major public health issue that the world is currently facing. The number of obese people in almost all age groups is increasing. Currently, the phenomenon of overweight and obesity in China is increasing year by year. According to literature reports, the proportion of overweight and obesity among adults aged 18 and above has reached a high level of 34.3% and 16.4%, respectively (2). In developed countries, the obesity rate is as high as over 30%, while in developing countries, the obesity rate is also showing a sharp upward trend. Obesity not only changes the appearance of the body, but also poses a serious threat to physical health to a certain extent (3). Obesity is a complex metabolic disease that affects almost all organ systems. Common obesity related diseases include type 2 diabetes, obstructive sleep apnea, multiple cancers (such as pancreatic cancer and gastrointestinal cancer), mental health conditions (such as depression and anxiety), liver and kidney diseases, osteoarthritis and cardiovascular diseases. At the same time, cardiovascular diseases are the main cause of death (4–6). Obesity is mainly a state of imbalance between energy intake and expenditure, leading to excessive accumulation of fat in the body. The current common understanding is that exercise leads to weight loss (7), but in reality, many people cannot persist, so the development of weight loss drugs has become a focus of current research.

However, the drugs currently available on the market for regulating blood lipids inevitably come with certain adverse reactions (8). Therefore, the development of natural, safe and harmless functional foods that can effectively regulate lipid metabolism disorders is particularly urgent and important.

In recent years, with the deepening of research on lipid-lowering drugs by researchers, hundreds of traditional Chinese medicines with lipid-lowering functions have been discovered (9). For example, Chinese herbal medicines such as lotus leaves, cassia seeds, hawthorn, Polygonum multiflorum, and Panax notoginseng have been proven to have significant lipid-lowering activity (10, 11). According to literature reports, the main ways of action of traditional Chinese medicine are to lower cholesterol, lower triglycerides, nourish the liver, and kidneys, strengthen the spleen and stomach, nourish blood and qi, and promote blood circulation to remove blood stasis. These remedies often contain a rich array of bioactive compounds such as polyphenols, polysaccharides, alkaloids, flavonoids, and terpenoids, which exert anti-obesity effects through diverse mechanisms (12–15). For instance, mulberry leaf has demonstrated anti-obesity and anti-hyperlipidemia activities by decreasing the Firmicutes/Bacteroidetes ratio, increasing SCFA production, and reducing lipid accumulation in the liver and adipose tissue (12). Rhizoma Polygonati, an herb-homology-food, also contains potential active compounds with therapeutic properties (16). However, traditional herbal medicines face challenges, including low content of bioactive components, poor aqueous solubility, limited bioavailability, and potential interactions with the gastrointestinal tract, which can hinder their therapeutic potential (17–19). Furthermore, some herbal ingredients may have potential toxic side effects (18, 20). According to literature reports, probiotics can produce cellulase, pectinase, xylanase, lipase, etc. (21), and use probiotics to ferment Chinese herbal medicine, thereby increasing the content of active ingredients in Chinese herbal medicine and enhancing its lipid-lowering effect. At present, Chinese herbal medicine, as a traditional treatment method, has the advantages of precise efficacy and minimal side effects, and is highly favored by people.

The concept of probiotic fermentation of herbal residues has emerged at the intersection of these two challenges, offering a sustainable approach to waste reduction while potentially yielding valuable therapeutic agents for obesity management. Probiotics, defined as live microorganisms that confer health benefits when administered in adequate amounts, have demonstrated potential in modulating gut microbiota composition and function, thereby influencing host metabolism. Based on extensive research, probiotics can regulate gut microbiota and improve symptoms of weak inflammatory response in the body; Traditional Chinese medicine has the advantages of precise regulation of lipid metabolism, minimal toxic side effects, easy availability of materials, and long-term use; And the two interact and complement each other. This review systematically examines current evidence on probiotic-fermented herbal residues for obesity management, focusing on the scientific basis, methodological approaches, efficacy demonstrated in experimental models, proposed mechanisms of action, and future research directions. By integrating traditional knowledge with modern biotechnology, this study provides a brief overview in order to provide reference for the causes and prevention of obesity.

## 2 Scientific basis of herbal residues and probiotic fermentation

### 2.1 Composition and potential of herbal residues

Traditional Chinese medicine industry is China's traditional competitive industry. In recent years, with the enhancement of people's health awareness and the rapid development of traditional Chinese medicine, Chinese herbal medicine and traditional Chinese patent medicines and simple preparations have been increasingly widely used. At the same time, a large number of wastes—Chinese medicine dregs have inevitably been produced. According to statistics, China discharges up to 10 million tons of traditional Chinese medicine residue annually (22). As is well known, traditional Chinese medicine residue has a high moisture content and abundant organic matter, making it highly susceptible to spoilage and deterioration. If not treated in a timely manner, it will have a serious impact on the environment. What's more serious is that if these residues are confiscated by criminals and used to manufacture fake drugs, it will cause huge losses to drug manufacturers and consumers (23).

Traditional Chinese medicine residue is a solid waste produced after extracting medicinal components from medicinal plant materials. After a single extraction, the residual amount of medicinal components in the traditional Chinese medicine residue is also significant, for example, the residual amount of flavonoids in Epimedium residue is greater than 40% (24). Traditional Chinese medicine residue contains abundant organic compounds such as polysaccharides, cellulose, vitamins, and crude protein, as well as trace elements. The transformation of these components into treasures in traditional Chinese medicine residue has achieved certain results (25). Adopting reasonable scientific methods and technologies to fully transform and utilize reusable resource-based substances, reducing the pressure and harm caused by organic waste such as traditional Chinese medicine residue in the pharmaceutical industry to the ecological environment.

### 2.2 Fundamentals of probiotic fermentation

Traditional Chinese medicine fermentation has long relied on wild natural bacterial strains, which have limitations such as diverse strains and uncertain functions. With the development of biotechnology, modern fermentation techniques tend to use specific single or composite strains for fermentation. As an important foundation for the production of fermented traditional Chinese medicine products, strain selection has become a focus of academic attention (26). At present, the microorganisms commonly used for fermentation of Chinese herbal medicine mainly include lactic acid bacteria, Bacillus subtilis, yeast, and mold. The commonly used strains in recent years are shown in Table 1. Lactic acid bacteria (such as Lactobacillus plantarum, Lactobacillus acidophilus, Lactobacillus reuteri, etc.) can produce various organic acids, lower the pH of the fermentation system, inhibit the growth of miscellaneous bacteria, and ensure the stability of fermented traditional Chinese medicine. In addition, they can also generate active substances such as antimicrobial peptides and short chain fatty acids, which are beneficial to host health (27). Bacillus subtilis (such as Bacillus subtilis, Bifidobacterium, Bacillus licheniformis, etc.) has strong tolerance and can grow and reproduce under high temperature conditions, making it suitable for certain high-temperature fermentation processes. This type of bacteria can also secrete various enzymes, which help to decompose complex substrates and improve fermentation efficiency (28–30). Yeasts (such as brewing yeast, Candida albicans, etc.) reproduce quickly and have high fermentation efficiency, making them suitable for rapid fermentation processes. Meanwhile, yeast can produce various flavor compounds, improving the taste and aroma of fermented traditional Chinese medicine (31, 32). Fungi (such as Aspergillus oryzae, Rhizopus, Aspergillus niger, Penicillium, etc.) can secrete extracellular enzymes such as proteases, hemicellulases, and cellulases to break down proteins and cellulose in traditional Chinese medicine, thereby improving fermentation efficiency and medicinal utilization (33–35). These microorganisms function as natural biocatalysts through their enzymatic systems that break down complex plant structures and convert constituents into more bioavailable forms.

**Table 1:** List of Chinese herbal fermentation strains.

Fermentation strain	Fermentation substrate	References
*Lactobacillus bulgaricus Bifidobacterium spp*	fenugreek seeds, aloe vera	(87)
*Lactobacillus acidophilus and Bacillus subtilis*	Honeysuckle-Cassia seeds extracts	(38)
*Lactobacillus plantarum*	Astragalus	(139)
*Lactobacillus acidophilus*	Lingzhi, Sanqi	(140, 141)
*Bifidobacterium breve*	Lingzhi	(140)
*Lactobacillus reuter*	Sanqi	(141)
*Bacillus licheniformis*	Largehead Atractylodes Rhizome	(30)
*Monascus*	kudzu root	(142)
*Yeast*	rhubarb	(143)
*Saccharomyces cerevisiae*	Ge Gen Qin Lian Tang	(144)
*Aspergillus oryzae*	Salvia miltiorrhiza	(72)
*Ganoderma lucidum Karst*	Ganoderma lucidum Karst	(73)

### 2.3 Fermentation techniques and methodological advances

The fermentation process can be conducted through various methodologies, including solid-state fermentation, liquid fermentation, and bidirectional fermentation, each offering distinct advantages for specific applications. Modern approaches often employ co-culture systems or enzyme-assisted fermentation to further enhance the efficiency and output of the biotransformation process (36). Comparison of different fermentation modes for fermenting traditional Chinese medicine residues is shown in Table 2.

**Table 2:** Comparison of fermentation techniques for herbal residues.

Fermentation technique	Advantages	Limitations	Applications in obesity management	References
Solid-State Fermentation	1. The process is relatively simple, the cost is low, and it is suitable for treating solid waste such as pharmaceutical residues 2. It can effectively maintain microbial activity and facilitate the production of abundant secondary metabolites 3. Commonly used in the production of feed additives or organic fertilizers to achieve resource recycling	1. The fermentation process is difficult to control, prone to contamination with miscellaneous bacteria, and has poor batch stability −1 2. The mass and heat transfer efficiency is low, and the fermentation cycle may be long. 3. The composition of the final product is complex and the standardization of quality is difficult	SHEIH et al. used Aspergillus niger for solid-state fermentation of Astragalus membranaceus, and found that the phenolic content and antioxidant activity in fermented Astragalus membranaceus were significantly higher than those in unfermented Astragalus membranaceus	(39, 145)
Liquid-State Fermentation	1. Easy to control conditions (temperature, pH, dissolved oxygen), high degree of automation, and good product uniformity 2. High yield, short cycle, suitable for large-scale production of active substances such as polysaccharides and flavonoids 3. Beneficial for subsequent extraction and purification processes	1. Generate a large amount of fermentation waste liquid, which may increase environmental protection costs 2. The requirements and energy consumption for the equipment (fermentation tank) are relatively high 3. The metabolites of some bacterial strains in liquid environments may differ from those in solid-state fermentation	1. Fermented hawthorn juice has been proven to have lipid-lowering and anti fatty liver effects The *in vitro* lipase inhibitory activity of fermented Shenhe Ling extract was significantly enhanced after fermentation 2. Li et al. used swamp red pseudomonas liquid fermentation to ferment astragalus, and the polysaccharide content of astragalus increased by 51.56% compared to the unfermented group. 3.Su et al. used FGM liquid fermentation to increase the content of Huangqi root, Huangqi polysaccharide, total flavonoids, and total saponins by 177.46%, 55.67%, and 68.59%, respectively	(146–149)
Microbial-Enzyme Synergistic Fermentation	1. High efficiency: Enzyme preparations can quickly destroy plant cell walls, release effective ingredients, and create favorable conditions for microbial utilization 2. Strong directionality: By adding specific enzymes, targeted active ingredients can be enriched or transformed (such as converting starch into resistant starch) 3. It can shorten fermentation time and improve conversion efficiency	1. Cost increase: The use of enzyme preparations has increased production costs 2. Complex process: It is necessary to optimize the order, proportion, and working conditions of enzyme and bacteria addition 3. Changes in enzymatic hydrolysis products may have uncertain effects on subsequent microbial fermentation	By utilizing enzymatic co fermentation, the starch in Bai Zhi residue can be converted into resistant starch (RS3), which can significantly regulate the gut microbiota of obese mice and improve metabolic disorders	(150)
Bidirectional Fermentation	1. Synergistic effect enhancement: The interaction between medicinal matrix (Chinese herbal residue) and medicinal fungi can promote fungal growth and transform medicinal ingredients, which may produce new active compounds 2. “Medicinal attraction” effect: Traditional Chinese medicine ingredients may guide the metabolic pathways of fungi, enabling them to produce more targeted functional ingredients	1. Complex mechanism: The interaction mechanism between bacteria and drugs is unclear, making research difficult 2. High substrate requirements: Different medicinal herbs have significant differences in their effects on fungal growth, and pairing combinations need to be screened and optimized 3. Lack of standardized processes and quality evaluation systems	The bidirectional solid-state fermentation of Ganoderma lucidum and ginseng significantly enhanced the immune regulatory activity of ginseng; The bidirectional liquid fermentation of Ginkgo biloba leaves and Ganoderma lucidum significantly increased the production of polysaccharides, triterpenoids, and total flavonoids, and enhanced antioxidant activity. The dual fermentation system of Monascus purpureus and Betula platyphylla can significantly increase the content of β–glucan and exhibit good lipid-lowering activity. The antioxidant activity of the dual fermentation product of Monascus purpureus and Dendrobium nobile is significantly improved	(151–155)
Co-cultivation fermentation	1. Simulating nature: A system with multiple microorganisms coexisting is closer to a natural fermentation state and has a richer metabolic network 2. Functional complementarity: Different bacterial strains can collaborate and complete complex transformations that cannot be achieved by a single strain, which may enhance efficacy	1. Unstable system: There is competition or antagonism between strains, making it difficult to maintain a stable population ratio 2. Process control is extremely difficult: it is difficult to accurately monitor and regulate the growth dynamics of each microorganism 3. Poor reproducibility is a major challenge facing industrialization at present	The fermentation products of complex microbial communities may affect energy metabolism and intestinal health through multiple targets and pathways. For example, fermented tea involving multiple microorganisms such as Aspergillus niger has been shown to exert anti obesity effects through the “gut microbiota metabolite fat thermogenesis” axis	(156, 157)

#### 2.3.1 Solid-state fermentation

Solid-state fermentation (SSF) represents one of the most common approaches for herbal residue biotransformation. In SSF, microorganisms grow on moist solid substrates in the absence of free water, creating conditions that often enhance enzyme production and metabolic activity compared to submerged fermentation (33). This method is particularly suitable for herbal residues as it mimics traditional fermentation processes while allowing for better control and standardization.

SSF of herbal residues typically involves inoculating the substrate with selected probiotic strains and maintaining controlled conditions of temperature, humidity, and aeration to optimize the fermentation process (37). The simplicity and low energy requirements of SSF make it particularly attractive for industrial applications. Research has demonstrated that SSF can significantly enhance the bioactive compound profile of various herbal residues, with studies reporting increases of 62%−275% in total polysaccharides, flavonoids, and saponins after fermentation (38). SHEIH et al. used solid-state fermentation of Astragalus membranaceus with Aspergillus niger for 3 days, successfully increasing the content of phenolic substances in Astragalus membranaceus, the antioxidant activity of fermented Astragalus membranaceus is significantly higher than that of unfermented Astragalus membranaceus (39).

#### 2.3.2 Enzyme-assisted probiotic fermentation

A notable advancement in fermentation methodology is the integration of enzymatic pretreatment with probiotic fermentation, creating a synergistic bioprocessing system. This approach combines specific enzymes such as cellulases, xylanases, and pectinases with probiotic strains to enhance the breakdown of plant cell walls and liberation of bioactive compounds (40).

The enzyme-assisted approach addresses a key limitation in herbal residue utilization—the robust plant cell walls that trap bioactive compounds. As noted in research on herbal residue valorization, “cellulase, xylanase, pectinase: directly and efficiently decompose plant cell walls, release intracellular nutrients, increase fermentation substrates, and improve the fermentation level” (41). This method simultaneously increases the yield of bioactive compounds and reduces potential toxicity through the transformation of toxic components.

#### 2.3.3 Bidirectional fermentation and co-culture systems

**Figure 1:**
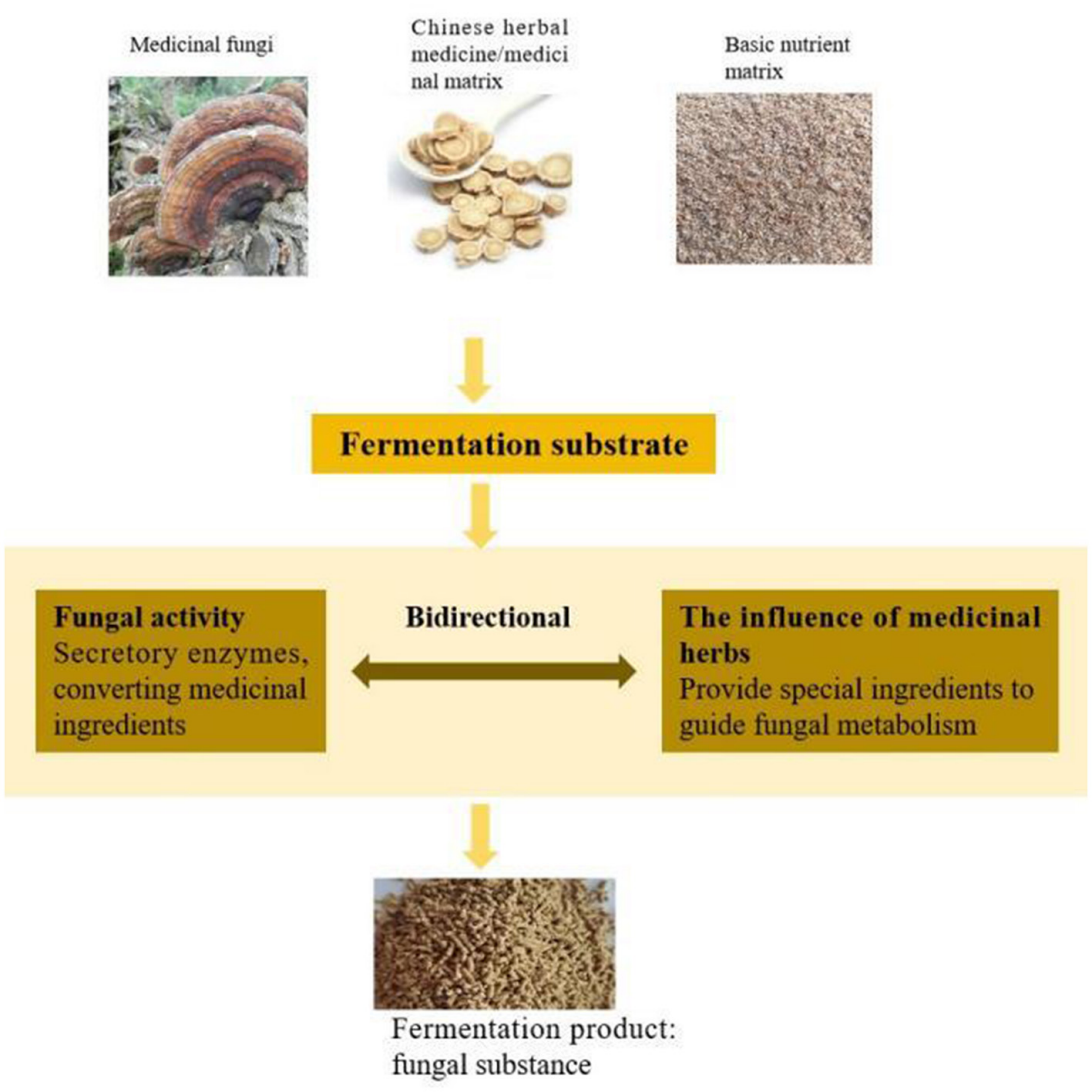
Schematic diagram of bidirectional fermentation.

Bidirectional fermentation represents an innovative approach where medicinal fungi are used to ferment herbal substrates, creating complex interactions between the fungus and the herbal compounds, abbreviated as bidirectional fermentation (Figure 1). This technique, pioneered by researchers like Zhuang Yi, employs a “three-layer optimization method” and “dynamic comparison of substrate, bacterial quality composition, and efficacy” to systematically evaluate and optimize the fermentation process (42). When studying the bidirectional fermentation products of yam and cordyceps militaris, Li et al. found that the fermentation process promotes the conversion of polysaccharides, cellulose and other carbohydrates in yam into polyphenols and saponins, while significantly enhancing its antioxidant capacity (43). The research conducted by Liu et al. showed that after bidirectional fermentation of Sophora flavescens and Radix Isatidis, the content of polysaccharides and inosine in the products significantly increased, and the resulting fermentation yeast showed strong anti-inflammatory effects (44). Research has shown that microorganisms such as Aspergillus and Penicillium can transform various traditional Chinese medicine components such as ginsenosides, playing a role in enhancing efficacy, reducing toxicity, and obtaining new compounds (45, 46). Therefore, the use of this bidirectional fermentation technology to develop traditional Chinese medicine residues can not only achieve green recycling of waste, but also open up new avenues for secondary development of traditional Chinese medicine, which can be said to have multiple benefits and significant economic benefits (47).

Similarly, co-culture systems utilizing multiple probiotic strains have demonstrated superior results compared to single-strain fermentations. With the expansion of cognition, multi strain collaborative fermentation has emerged in the field of traditional Chinese medicine fermentation. This method combines the advantages of stable fermentation process and strong resistance to miscellaneous bacteria, and can utilize the different metabolic characteristics between strains to enrich the chemical composition of the final product (48). For instance, Professor Wei Ying's team from Beijing University of Agriculture developed a ^**^dual-bacteria co-fermentation system^**^ using *Bacillus subtilis* and *Lactobacillus acidophilus* to ferment honeysuckle-Cassia seed extracts. In this system, *Bacillus subtilis* acts as a bio-wall-breaking engine to efficiently disintegrate the dense plant tissue structure of honeysuckle-Cassia seeds, releasing deep active substances; while *Lactobacillus acidophilus* further functions as a bio transformer, converting macromolecular substances into small molecular components that are easily absorbed and highly active (38). Jin et al. (49, 50) used a mixture of Bacillus subtilis, Candida albicans, and Lactobacillus acidophilus to ferment and modify Sijunzi Tang, significantly improving its nutritional value and crude polysaccharide yield. Qiao et al. (51) used Enterococcus faecalis and Lactobacillus plantarum to ferment Astragalus membranaceus, and constructed a corresponding quality control platform based on PacBio single molecule real-time sequencing technology. Multi strain collaborative fermentation is an important direction that is in line with traditional Chinese medicine and has advantages in efficiency and utilization. However, the complexity of its system poses challenges for process control and mechanism research. The breakthrough point in the future lies in the application of real-time monitoring technology to dynamically analyze the fermentation process and obtain solid scientific basis (33).

## 3 Preparation of probiotic-fermented herbal residues

The preparation of probiotic-fermented herbal residues involves a systematic process that transforms raw herbal waste into a value-added product with potential health benefits. This transformation requires careful consideration of herbal residue selection, probiotic strain combination, and fermentation parameters to optimize the final product's efficacy.

### 3.1 Herbal residue selection and pretreatment

Herbal residues used in fermentation typically originate from various Chinese medicine formulations. For instance, residues from Jianweixiaoshi tablets (a digestant) have been successfully utilized in several studies (52, 53). These residues retain substantial nutritional value and bioactive compounds, including crude fiber, crude protein, crude fat, minerals, and residual active phytochemicals (54). The pretreatment process generally involves crushing or grinding the residues to increase surface area, followed by moisture adjustment to create an optimal environment for microbial growth. Some studies have also employed sterilization to eliminate competing microorganisms, though this is not universally practiced.

### 3.2 Probiotic strain selection and combination

The selection of appropriate probiotic strains is critical to the success of the fermentation process. Researchers typically employ strains with demonstrated functional properties relevant to the intended application. These strains are often used in **combination formulations** to leverage their complementary capabilities. For example, one study reported a four-strain combination consisting of *Bacillus subtilis* DC002, *Aspergillus oryzae* DC008, *Lactobacillus rhamnosus* CLT09, and *Bifidobacterium longum* NCC2705, which demonstrated superior fermentation outcomes compared to single-strain approaches (52).

### 3.3 Fermentation process and parameters

The fermentation process typically involves solid-state fermentation, where microorganisms grow on moist solid substrates in the absence of free water. This approach offers advantages such as higher product concentration, lower energy requirements, and reduced wastewater generation compared to submerged fermentation. Several critical parameters influence the fermentation outcome:

Water content: Optimal moisture levels typically range between 50%−80%, depending on the specific herbal residue and probiotic strains used (54). Higher moisture content (70%−80%) is generally preferred for bacterial fermentation, while fungal strains often perform better at lower moisture levels (50%−60%) (55–57).

Inoculation density: Common inoculation rates range from 5%−20% of the dry weight of herbal residues when using probiotic cultures with concentrations of 10^−7^-10^−8^ CFU/ml (58, 59).

Temperature: The optimal temperature varies according to the probiotic strains used, typically ranging from 20 °C−37 °C. Fungal strains such as *Aspergillus oryzae* generally prefer lower temperatures (25 °C−30 °C), while bacterial strains like *Bacillus subtilis* and *Lactobacillus* species thrive at higher temperatures (30 °C−37 °C) (54, 60).

Fermentation duration: The process may last from 2 days to 35 days, depending on the specific goals and microorganisms involved (54). Shorter fermentation times (2–5 days) are typically used when targeting high probiotic viability, while longer durations (15–35 days) may be employed to enhance bioactive compound transformation or fiber degradation (55, 61).

pH control: The initial pH is often adjusted to neutral or weakly acidic conditions (pH 5.5-7.0) using buffers, though some strains can adapt to a wider pH range (62).

Advanced fermentation strategies sometimes employ multi-stage processes to optimize outcomes. This sophisticated approach aims to maximize the release and transformation of bioactive compounds while enhancing their bioavailability.

## 4 Mechanisms of action in obesity management

Probiotic-fermented herbal residues exert their anti-obesity effects through multiple interconnected mechanisms, primarily mediated by the gut-microbiota-liver axis (63). These mechanisms involve modulation of gut microbiota composition, enhancement of gut barrier function, production of bioactive metabolites, and regulation of host metabolic pathways.

### 4.1 Modulation of gut microbiota composition

The intestinal microbiome plays a fundamental role in energy homeostasis, and its dysbiosis has been strongly associated with obesity development. Probiotic-fermented herbal residues help restore a healthy microbial ecosystem through several actions:

Fermented traditional Chinese medicine, under the transformation of microorganisms, can effectively improve the bioavailability of active ingredients and generate novel metabolites with biological activity; These products regulate and maintain the balance of intestinal microbiota through a multi-target synergistic mechanism (64). Research has shown that probiotic fermented traditional Chinese medicine and its metabolites have a significant improvement effect on the gut microbiota. For example, Liu Shen Qu fermented with probiotics can enhance gastrointestinal motility by regulating the secretion of gastrointestinal hormones, effectively adjusting the structure of gut microbiota, improving functional dyspepsia, and reducing related inflammatory reactions (65). The anthocyanins produced by hawthorn fermentation can selectively promote the proliferation of beneficial bacteria such as bifidobacteria and inhibit the growth of pathogenic bacteria such as Escherichia coli in the digestive tract, thereby optimizing the composition of the microbial community; This process also reduces liver lipid accumulation by enhancing fatty acid oxidation, inhibiting the expression of key enzymes involved in fat synthesis (66). Another study compared the therapeutic effects of Sijunzi Tang and its fermented preparation (inoculated with Lactobacillus plantarum) on antibiotic induced dysbiosis in mice with diarrhea. It was found that fermented Sijunzi Tang can promote intestinal development, increase villus height, significantly improve diarrhea symptoms, and inhibit the growth of harmful bacteria (67). A key mechanism through which fermented herbal residues exert their anti-obesity effects is by remodeling the gut microbiota. The fermentation process enhances the prebiotic effects of herbal residues, making them more accessible to beneficial gut bacteria. This creates a positive feedback loop where the fermented product simultaneously delivers probiotics and optimized prebiotics to the gastrointestinal tract.

### 4.2 Production of bioactive metabolites

The synergistic action of probiotics and herbal components during fermentation leads to the generation and transformation of various bioactive metabolites that influence host metabolism:

Traditional Chinese medicine residue contains various active ingredients, which give it special medicinal value and recycling prospects. According to research, unfermented traditional Chinese medicine residue still retains 30% to 50% of effective active substances. After fermentation treatment, the content of some active ingredients can reach 1 to 4 times that of the original (68). By screening specific strains, targeted enhancement of active ingredients can be achieved, thereby improving the biotransformation efficiency during the fermentation process. Using endophytic fermentation to treat Epimedium herb residue can increase the total flavonoid content to a maximum of 16.923 mg/g, with an increase of 15.32% (69). After fermentation, the content of total glycosides, rosmarinic acid, chlorogenic acid, and total polyphenols in Niuzhi residue increased to 1.351%, 2.733%, 12.323%, 21.604%, and 0.16%, respectively (70). By fermenting Codonopsis pilosula residue with Lactobacillus plantarum, the content of secondary metabolites significantly increased. Flavonoids, lignans, alkaloids and their derivatives, organic acids and their derivatives increased by 1.13 times, 1.04 times, 1.01 times, and 1.16 times, respectively (71).

In the process of microbial transformation, fermentation strains use their enzyme system to adjust the structure of traditional Chinese medicine components, promoting the decomposition, transformation, or synthesis of active new substances from existing chemical components (26). After fermentation by Aspergillus oryzae, the phenolic and flavonoid components in Danshen underwent structural modifications, further optimizing its pharmacological properties (72). After using Ganoderma lucidum to ferment Tripterygium wilfordii, the peak intensity of Triptolide in the mass spectrum was about 2.15 times that before fermentation; the peak intensity of the mass spectrum of Tripterygium wilfordii Hook. f. was increased to about 5.15 times that before fermentation, and the peak intensity of the mass spectrum of resveratrol was increased to about 7.25 times that before fermentation. These changes in composition are closely related to the attenuated and enhanced effects of fermentation products (73, 74). Fermentation with Lactobacillus plantarum KCCM 11613P converts ginsenosides Rb2 and Rb3 in red ginseng extract into ginsenosides Rd, increasing the titratable acidity, viable cell count, and antioxidant activity of the fermentation broth (75).

Some traditional Chinese medicines have certain toxicity and irritability (76, 77). After microbial fermentation treatment, the relevant toxic or irritating components can be decomposed or transformed, thereby reducing or eliminating adverse reactions and improving the taste of the drug (78). For example, Jiang et al. (79) used Ganoderma lucidum to ferment Chuanwu and found that the content of total alkaloids and ester alkaloids decreased after fermentation. Peng et al. (80) evaluated the toxicity changes of “Tremella fuciformis” during the fermentation process through acute toxicity tests, and the results showed that the fermentation of Tremella fuciformis could significantly reduce the toxicity of Malvaceae. In addition, some strains used for fermentation also exhibit attenuated effects *in vivo*. Mohd Redzwan et al. (81) found that continuous intake of probiotics can significantly affect the metabolism of aflatoxins, helping to prevent the risk of exposure to aflatoxins in the diet. At present, the relevant detoxification mechanisms are not fully understood and require further in-depth research.

### 4.3 Regulation of lipid metabolism

Probiotic-fermented herbal residues demonstrate significant regulatory effects on key metabolic pathways involved in lipid homeostasis.

#### 4.3.1 Weight reduction and fat accumulation

Research has shown that when using microorganisms to ferment traditional Chinese medicine, the metabolites produced can break down the active ingredients into smaller molecules of active substances. This not only helps accelerate the absorption of drugs by the human body, but also expands the metabolic pathways of microorganisms themselves (82), thereby enhancing their efficacy in antioxidant, anti-inflammatory, anti neurodegenerative, and anti obesity aspects (83, 84). Various traditional Chinese medicines have shown lipid-lowering effects, such as red yeast rice, which is a fermented product of Monascus on rice and can lower blood lipids by inhibiting cholesterol synthesis. Zhang (85) used Ganoderma lucidum, Gynostemma pentaphyllum, Cassia seed, and Tartary buckwheat as fermentation substrates, adopted liquid deep fermentation technology, and further enhanced its original lipid-lowering effect by optimizing the fermentation process. The use of composite probiotics for synergistic fermentation of Panax notoginseng, licorice, and astragalus can significantly improve the extraction efficiency of polysaccharides, increasing their yield by 2.5 times; The polysaccharide components contained in the obtained fermentation broth have been confirmed to have the function of regulating blood lipids (86).Experimental studies have consistently demonstrated that probiotic-fermented herbal residues significantly reduce body weight gain and fat accumulation in obese animal models. In a study investigating the effects of nanoemulsions containing probiotics (*Lactobacillus bulgaricus, Bifidobacterium*) and local herbs (fenugreek seeds, aloe vera) on high-fat diet-induced obese rats, the combination treatment exhibited protective effects on metabolic organs and significantly reduced obesity parameters (87). Histological examinations further confirmed that the fermented preparations ameliorated adipocyte hypertrophy and irregular morphology in fat tissues.

#### 4.3.2 Improvement in lipid profile and liver function

In recent years, research on lipid-lowering drugs has continued to advance, and hundreds of varieties with lipid-lowering effects have been discovered in traditional Chinese medicine (9). Multiple studies have confirmed that traditional Chinese medicines such as lotus leaves, Gynostemma pentaphyllum, Cassia seed, hawthorn, Polygonum multiflorum, and Panax notoginseng have significant lipid-lowering effects (10, 11). These drugs mainly exert their effects through mechanisms such as regulating blood lipids, nourishing the liver and kidneys, invigorating the spleen and promoting blood circulation, resolving phlegm and promoting diuresis (88). Their active ingredients include polysaccharides, unsaturated fatty acids, saponins, flavonoids, polyphenols, alkaloids, and anthraquinones (89). The above active ingredients lower blood lipids through various mechanisms, mainly including inhibiting the absorption, synthesis, and release of endogenous lipids, enhancing the antioxidant defense system, and increasing the level and activity of high-density lipoprotein cholesterol (HDL-C) (90). Alisma alcohol extract can effectively improve liver fat metabolism, and its mechanism of action is to reduce acetyl CoA required for cholesterol synthesis, thereby inhibiting lipid accumulation in the liver at the source (91). Simultaneously, it can inhibit oxidative stress and alleviate energy metabolism abnormalities caused by high-fat diet. Fermented herbal residues consistently demonstrate lipid-lowering effects and hepatoprotective properties in experimental models of obesity (92). The combination of cholesterol lowering probiotics and anthraquinone from Cassia seed can synergistically upregulate the expression of lipid metabolism enzymes (such as cholesterol 7 α–hydroxylase and low-density lipoprotein receptor) and activate PPAR—α, while inhibiting the expression of some rate limiting enzymes and PPAR—γ, regulating lipid metabolism in the liver through multiple pathways to reduce blood lipids (93). Research on a tangerine peel-based herbal formula (Tanshi-Tiaoti Decoction) demonstrated significant improvements in fasting glucose levels, serum triglycerides, and hepatic steatosis in high-fat diet-induced metabolic syndrome mice. The fermented formulation restored normal bile acid metabolism through modulation of the gut microbiota, particularly by reducing the Firmicutes/Bacteroidetes ratio and enhancing the production of hyodeoxycholic acid (HDCA), which activates TGR5 receptors and promotes beige adipocyte browning (94). Researchers have attempted to use probiotic fermentation technology to treat medicinal materials. This technology can effectively increase the yield and bioavailability of active ingredients, thereby enhancing the lipid-lowering effect. Therefore, traditional Chinese medicine therapy maybe become an important research direction and treatment option for addressing obesity and hyperlipidemia.

### 4.4 Enhancement of gut barrier function and reduction of inflammation

#### 4.4.1 Microbiota-mediated mechanisms

The microbiota plays a central role in the anti-obesity effects of fermented herbal residues through multiple pathways. Microbial transformation can generate new bioactive ingredients, improve the odor of traditional Chinese medicine, enhance drug efficacy, and reduce adverse effects. Short chain fatty acids (SCFAs) are important metabolites of gut microbiota (95, 96). Among them, acetic acid, propionic acid, and butyric acid are the most common short chain fatty acids. These metabolites play a central role in regulating host lipid metabolism through multiple pathways and receptors, and have become a hot research topic (97). Many traditional Chinese medicines can mediate their anti obesity effects by regulating the gut microbiota, effectively inhibiting fat accumulation. The key mechanisms include upregulating the production of short chain fatty acids (SCFAs), promoting the release of glucagon like peptide-1 (GLP-1) through the gut brain axis pathway, and regulating the process of bile acid metabolism (98). The polysaccharide active ingredients contained in various traditional Chinese medicines can exert antioxidant effects by increasing the levels of short chain fatty acids (SCFAs), thereby promoting lipid metabolism. For example, polysaccharides from hawthorn (99), Polygonatum sibiricum (100, 101), yam (102), dendrobium (103), cordyceps (104), and lingzhi (105) all have such effects. Sea buckthorn polysaccharides reduce lipid accumulation by regulating the structure of gut microbiota: they can reduce the abundance of Desulfovibrio while increasing the abundance of beneficial bacteria such as Akkermansia and Bacteroides. This structural adjustment enhances the expression of genes related to lipid breakdown and increases the production of short chain fatty acids (SCFAs), ultimately inhibiting lipid accumulation (106, 107).

**Figure 2:**
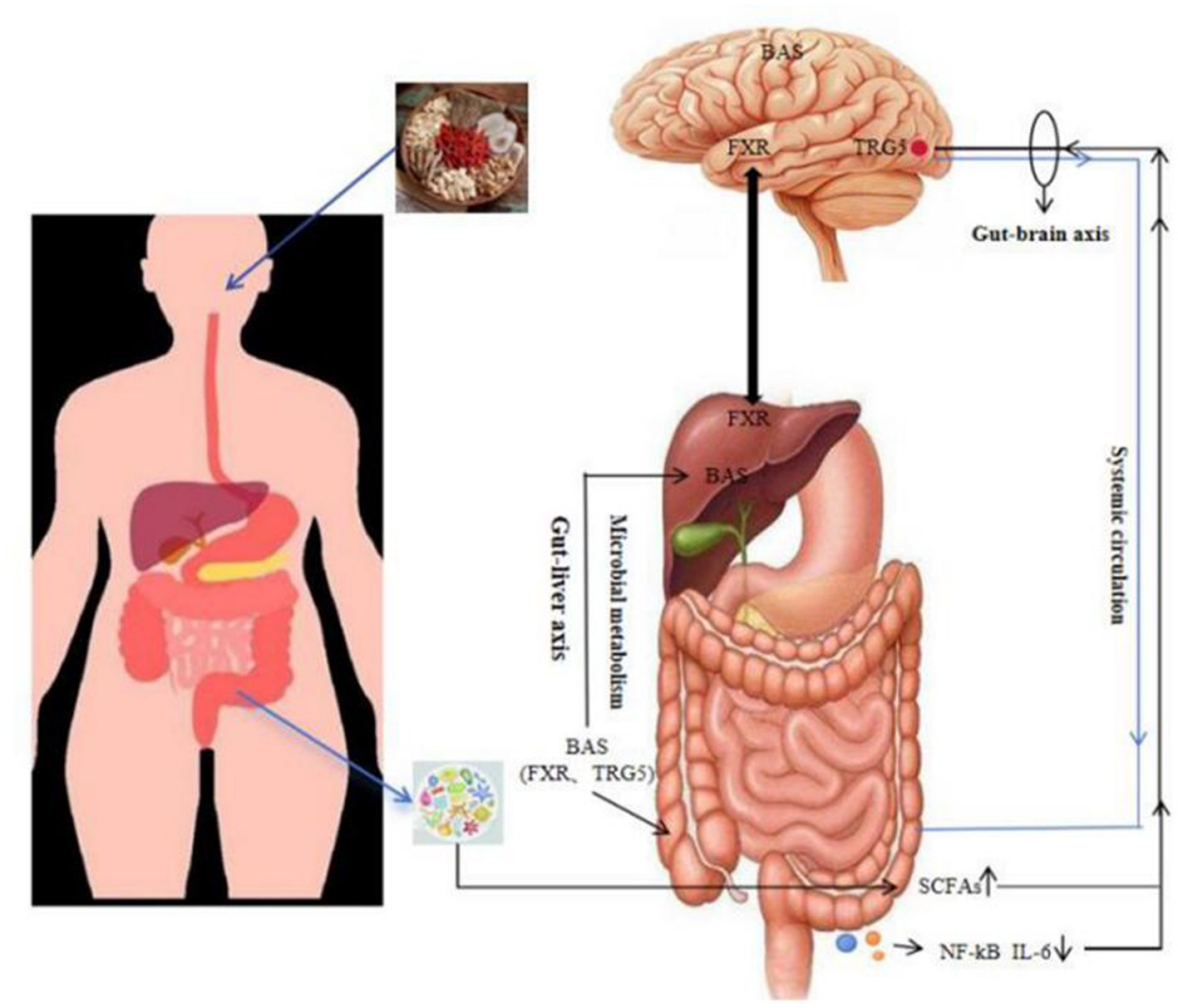
Lipid-lowering mechanism of traditional Chinese medicine based on intestinal flora.

Bile acids can also dissolve and promote the absorption of dietary lipids and fat soluble nutrients. The bile acid (BA) in the intestine produces FGF19/15 by binding to the FXR receptor, which reaches the hypothalamus via the gut brain axis, thus regulating glucose metabolism. With the appetite inhibition and energy regulation of FGF19, the prevention and treatment of metabolic diseases such as type 2 diabetes and obesity can be achieved (108). Triterpenoids in Alisma can increase the metabolism and transport of BA and enhance cholesterol breakdown by activating FXR receptors (109, 110). Eugenol increases the abundance of *Lactobacillus acidophilus*, enhances intestinal barrier function and BA metabolism levels to regulate lipid metabolism (111). Research has confirmed that browning of white adipose tissue is an important pathway for reducing lipid accumulation (112). Traditional Chinese medicines such as Poria cocos, Coix seed (113), and Danshen (114) promote this process through multidimensional mechanisms, including inhibiting adipocyte autophagy, regulating insulin resistance, and improving inflammation. These mechanisms work together on relevant signaling pathways, effectively promoting browning of white adipose tissue in the body, ultimately achieving significant anti obesity effects. In summary, traditional Chinese medicine exerts its lipid-lowering effects effectively through the interaction of multiple pathways, as shown in Figure 2.

#### 4.4.2 Metabolic and inflammatory pathways

Beyond microbiota modulation, fermented herbal residues influence obesity through direct metabolic and anti-inflammatory effects. Research has shown that the main cause of obesity and hyperlipidemia is the synthesis of excessive lipids, and traditional Chinese medicine mainly intervenes in obesity related metabolic diseases through lipid metabolism (115). Lactobacillus plantarum dy-1 and yeast were used as fermentation strains to investigate the mechanism of LFBE in inhibiting 3T3-L1 preadipocyte differentiation, downregulating the expression of PPAR-γ, C/EBP-α, and SREBP-1c genes related to adipocyte differentiation, and inhibiting adipocyte differentiation (116). Li discovered that the fermentation broth of Ge Gen Qin Lian Tang reduces serum total cholesterol and triglyceride levels, and regulates glucose and lipid metabolism (117). Lactic acid bacteria fermentation of Moringa leaves reduces liver lipid accumulation, lowers glucose metabolism and oxidative stress, and inhibits inflammation (118). *Lactobacillus plantarum*.

BL2 fermented garlic extract reduces the concentration of triglycerides and total cholesterol in serum, downregulates PPAR-γ, C/EBP-α MRNA and protein expression of SREBP-1c, FAS, and SCD-1 inhibit adipogenesis (119). Research has shown that hawthorn can inhibit ER stress and PPAR-γmediated lipid synthesis in liver cells of high-fat diet rats, thereby exerting a protective effect on obesity related hepatic steatosis (120).

Damaged intestinal barrier function can cause intestinal bacteria or their harmful metabolites to translocate into the bloodstream, leading to systemic inflammation, which is the core pathogenic mechanism of various human diseases such as obesity (121). Therefore, anti-inflammatory or regulation of related microbiota can be a potential strategy for improving obesity. Multiple studies have shown that active ingredients in traditional Chinese medicine, such as ginsenosides (122), saponins from Panax notoginseng (123), loganin (124), saikosaponin (125), and baicalin (126), have been proven to effectively repair the intestinal mucosal barrier, reduce inflammatory factors and intestinal endotoxins, and thereby improve intestinal metabolic imbalance caused by obesity. The key mechanism of action of probiotic fermented herbal residue in obesity management is shown in Table 3. These multifaceted mechanisms work synergistically to address various aspects of obesity pathophysiology, positioning probiotic-fermented herbal residues as a promising multi-target approach to weight management and metabolic health improvement.

**Table 3:** Key mechanisms of action of probiotic-fermented herbal residues in obesity management.

Mechanism category	specific actions	Outcomes	References
Microbiota modulation	Increase in beneficial bacteria (*Bifidobacterium, Lactobacillus*)	Improved microbial diversity, reduced inflammation	(63)
	Decrease in harmful bacteria (*Lachnospiraceae bacterium_*28-4)	Reduced endotoxin production, improved metabolic parameters	(63)
Metabolic Regulation	Enhanced SCFA production	Improved satiety signaling, enhanced energy expenditure	(158)
	Activation of AMPK pathway	Improved lipid and glucose homeostasis	(158)
	Reduction of hepatic TG and TC	Improved liver function, reduced steatosis	(63)
Barrier & Immune Function	Strengthened intestinal tight junctions	Reduced metabolic endotoxemia	(158)
	Increased T regulatory cells	Attenuated chronic inflammation	(158)

## 5 Experimental evidence

The anti-obesity effects of probiotic-fermented herbal residues have been investigated through a range of experimental approaches, including *in vitro* studies, animal models, and preliminary human trials. The accumulating evidence provides insights into the efficacy and potential applications of this innovative strategy.

### 5.1 *In vitro* studies

*In vitro* investigations have primarily focused on characterizing the biochemical changes occurring during fermentation and assessing the biological activities of the resulting products: Enhanced bioactive compound content: Studies have demonstrated that fermentation significantly increases the concentration of bioactive compounds in herbal residues. For instance, fermentation of Jianweixiaoshi tablet residues with a four-strain combination (*Bacillus subtilis* DC002, *Aspergillus oryzae* DC008, *Lactobacillus rhamnosus* CLT09, and *Bifidobacterium longum* NCC2705) resulted in notable improvements in antioxidant capacity and increased reducing sugar and protein content (52). Additionally, increased antioxidant activity has been consistently observed, which may contribute to reducing oxidative stress associated with obesity. These *in vitro* findings provide foundational evidence for the transformation of herbal residues into bioactive forms through probiotic fermentation, justifying further investigation in biological systems.

### 5.2 Animal studies

Animal models, particularly high-fat diet (HFD)-induced obese mice, have been extensively used to evaluate the anti-obesity effects of probiotic-fermented herbal residues: Body weight and adiposity: A comprehensive study investigating TCMP (traditional Chinese medicine and probiotics) in HFD-fed mice reported a 10.894% reduction in final body weight compared to untreated HFD controls. More notably, TCMP administration resulted in significant reductions in specific fat depots: 13.83% decrease in epididymal fat, 3.99% reduction in mesenteric fat, and 15.18% decline in inguinal fat mass. In addition, histological analysis further confirmed that TCMP can significantly improve adipocyte hypertrophy caused by HFD and improve the results of glucose tolerance testing. Long term consumption of high-fat foods often leads to an imbalance in liver lipid metabolism. This study found that TCMP supplements can effectively reduce the increase in liver weight caused by HFD in mice, and significantly reduce triglyceride (TG) and total cholesterol (TC) levels. More importantly, after TCMP treatment, liver slices showed a decrease in both the number and size of fat droplets, indicating that TCMP helps restore the normal lipid metabolism state of the liver. 16S rRNA sequencing analysis revealed that TCMP treatment significantly increased gut microbiota α-diversity (Observed features and Chao1 index) and altered β-diversity in HFD-fed mice−9-10. The results indicate that TCMP can significantly enhance the diversity of gut microbiota, particularly by increasing the number of beneficial bacteria such as *Bifidobacterium* and *Akkermansia*, while reducing the proportion of potentially harmful bacteria in the V*errucomicrobia phylum* (63). Another study investigating fermented Jianweixiaoshi tablet residues in spleen-deficient mice demonstrated significant immunomodulatory effects, including increased levels of IL-2, IL-4, and interferon-γ (IFN-γ), suggesting enhancement of host immune function (53). This finding is particularly relevant given the chronic low-grade inflammation associated with obesity.

### 5.3 Human studies and clinical trials

While most evidence currently derives from preclinical studies, preliminary human data is beginning to emerge: Preclinical studies and human pilot studies suggest that PFHR can lead to modest reductions in body weight, waist circumference, and improvements in metabolic parameters (127). These effects are often attributed to enhanced satiety, reduced fat absorption, improved glucose metabolism, and attenuated low-grade inflammation (128). Inflammation is a key pathophysiological feature of obesity, with interactions between adipocytes and cancer cells stimulating pro-inflammatory cytokine upregulation (129). Despite the promising potential, the clinical evidence for PFHR in obesity management is still emerging. While some randomized controlled trials (RCTs) on probiotics, prebiotics, and synbiotics have shown benefits for anthropometric, cardiometabolic, and inflammatory markers, the specific effects of probiotic-fermented herbal residues are less extensively studied in large-scale trials (130, 131). For example, a systematic review found that probiotics may reduce body weight and fat percentage, particularly with multi-strain interventions and higher daily doses (132). Another systematic review and meta-analysis indicated that herbal medicines can improve lipid profiles and glycemic control in individuals with obesity and metabolic syndrome (133). However, variability exists in study designs, probiotic strains, herbal substrates, and outcome measures, making direct comparisons challenging (134, 135). Though these preliminary human findings are promising, more rigorously controlled clinical trials with larger sample sizes and longer follow-up periods are needed to establish definitive efficacy and safety profiles.

### 5.4 Methodological limitations and knowledge gaps

Critical evaluation of the literature reveals important limitations. Heterogeneity of interventions complicates cross-study comparisons, as studies employ diverse herbal sources, probiotic combinations, and fermentation conditions (128). Mechanistic inference remains largely correlational—while associations between microbiota changes and metabolic improvements are documented, causal pathways require confirmation through intervention studies such as fecal microbiota transplantation Study design quality in animal research often lacks blinding, randomization descriptions, or sample size justifications. Furthermore, the complexity of microbiota–host interactions and variability in TCM standardization represent fundamental challenges that addressed before clinical translation (128). These limitations underscore the need for cautious interpretation and highlight priorities for future research: multi-omics approaches, standardized protocols, and rigorous randomized controlled trials.

## 6 Outlook

The circular utilization of traditional Chinese medicine residue waste is crucial for promoting the green development of the traditional Chinese medicine industry and environmental protection. The traditional treatment methods for such large amounts of biological waste have problems such as high cost, heavy pollution, and low resource utilization rate. The current research and practical hotspots have shifted toward using pretreatment techniques to destroy its complex structure, and combining it with biological fermentation technology for efficient and high-value comprehensive utilization. The fermentation of herbal residues using probiotics represents an innovative approach that addresses two significant challenges simultaneously: the need for effective obesity treatments and the sustainable utilization of herbal medicine byproducts. This review has summarized compelling evidence that probiotic fermentation enhances the bioactive profiles, bioavailability, and therapeutic efficacy of herbal residues against obesity and its metabolic complications. The anti-obesity effects of these fermented preparations operate through multiple complementary mechanisms, including gut microbiota modulation, bile acid metabolism regulation, inflammatory pathway modulation, and enhancement of intestinal barrier function. For example, studies have shown that oligosaccharides in Rehmannia glutinosa can significantly regulate gut microbiota. Its special structure can resist gastric enzyme digestion, selectively promote the proliferation of probiotics after reaching the intestine, and thus play a role in regulating lipid metabolism (136). Puerarin (137) can directly act on host cells, alter the integrity of intestinal epithelial cells in obese mice, and prevent obesity phenotype. Flavonoids can protect the intestinal epithelial cell barrier, reduce the absorption of microbial metabolites such as endotoxins, and promote the proliferation of probiotics. Has significant lipid-lowering effect (138). The integration of modern fermentation technologies with traditional herbal knowledge creates a powerful platform for developing novel, effective, and sustainable interventions for obesity management.

Despite the promising results, several challenges impede the widespread application of fermented herbal residues in obesity management:

I. Mechanistic elucidation: While efficacy has been demonstrated in experimental models, the precise molecular mechanisms and active metabolites responsible for the anti-obesity effects require further characterization.
II. Standardization issues: The quality control and standardization of fermentation processes present significant challenges, as slight variations in conditions can substantially alter the metabolic output and therapeutic properties.
III. Strain selection optimization: Systematic approaches for selecting optimal probiotic strains for specific herbal residues are needed, as current methods often rely on empirical screening rather than predictive understanding.
IV. Stability and shelf-life: The long-term stability of bioactive compounds in fermented herbal residues and their shelf-life under various storage conditions require comprehensive evaluation.

Future research should focus on elucidating the precise mechanisms of action, optimizing fermentation processes, conducting rigorous human trials, and developing personalized approaches based on individual microbiome profiles. In additon, the need for Good Manufacturing Practice guidelines to ensure product quality and safety. The challenge of consistent production given variations in raw herbal materials and fermentation dynamics. And the potential role of metabolomics in defining a consistent chemical profile (a “fingerprint”) for quality control. The need for regulatory-grade toxicology studies (e.g., repeated-dose toxicity, genotoxicity) before human trials can proceed. As the field advances, probiotic fermented herbal residues have certain prospects as a means of promoting sustainable development in herbal production.

## References

[1] Baer DJ Dalton M Blundell J Finlayson G Hu FB. Nuts, energy balance and body weight. Nutrients. (2023) 15:e1162. doi: 10.3390/nu15051162 36904160 PMC10004756

[2] Pan J. Research progress on the harm and countermeasures of childhood obesity. J Shandong Nor Univ. (2023) 38:97–121.

[3] Heymsfield SB Wadden TA. Mechanisms, pathophysiology, and management of obesity. N Engl J Med. (2017) 376:254–66. doi: 10.1056/NEJMra1514009 28099824

[4] Kim MS Kim WJ Khera AV Kim JY Yon DK Lee SW . Association between adiposity and cardiovascular outcomes: an umbrella review and meta-analysis of observational and Mendelian randomization studies. Eur Heart J. (2021) 42:3388–403. doi: 10.1093/eurheartj/ehab454 34333589 PMC8423481

[5] Marie-Eve P Paul P Isabelle L Jean-Pierre D. Overview of epidemiology and contribution of obesity and body fat distribution to cardiovascular disease: an update. Prog Cardiovasc Dis. (2018) 61:103–13. doi: 10.1016/j.pcad.2018.06.004 29964067

[6] Koliaki C Liatis S Kokkinos A. Obesity and cardiovascular disease: revisiting an old relationship. Metabolism. (2019) 92:98–107. doi: 10.1016/j.metabol.2018.10.011 30399375

[7] Wang YH Yu W. Effects of precision exercise instruction supported by cardiopulmonary exercise testing on blood lipids, BMI and exercise levels in obese patients. Guide Chin Med. (2023) 21:72–4. doi: 10.15912/j.cnki.gocm.2023.31.011

[8] Arvanitis M Lowenstein CJ. Dyslipidemia. Ann Intern Med. (2023) 176:81–96. doi: 10.7326/AITC202306200 37307585

[9] Li HM Xie XY Chen ZH. Research progress in mechanism of traditional chinese medicine for lowering blood lipid. Strait Pharmaceutical Journal. (2021) 33:37–40.

[10] Li Man WX Li Xiaomei LI Qunwei. Overview about the regulatory function of Chinese Melicinal Herb on senum lipids. Med Rev. (2010) 16:1889–991.

[11] Men WH. Compound design of Chinese herbal medicineadditive for lowering cholesterol and its effect on thebiochemical index and egg quality of laying hens. Kunming Univ Sci Technol. (2016).

[12] Li R Wang J Liu J Li M Lu J Zhou J . Mulberry leaf and its effects against obesity: A systematic review of phytochemistry, molecular mechanisms and applications. Phytomedicine. (2024) 128:155528. doi: 10.1016/j.phymed.2024.155528 38555774

[13] Das G Ortega LAJ Gonçalves S Heredia JB Pereira MdLG Romano A . Anti-obesogenic effects of plant natural products: A focus on Korean traditional foods. Trends Food Sci Technol. (2024) 148:104470. doi: 10.1016/j.tifs.2024.104470

[14] Hussain A Bose S Wang J Yadav MK Mahajan GB Kim H. Fermentation, a feasible strategy for enhancing bioactivity of herbal medicines. Food Res Int. (2016) 81:1–16. doi: 10.1016/j.foodres.2015.12.026

[15] Alhamoud Y Abudumijiti T Wu J Lu L Zhao M Luo X . Stimulation of non-shivering thermogenesis by bioactive compounds: A focus on gut microbiota-mediated mechanisms. Trends Food Sci Technol. (2024) 154:104779. doi: 10.1016/j.tifs.2024.104779

[16] Mu C Sheng Y Wang Q Amin A Li X Xie Y. Potential compound from herbal food of *Rhizoma Polygonati* for treatment of COVID-19 analyzed by network pharmacology: viral and cancer signaling mechanisms. J Funct Foods. (2021) 77:104149. doi: 10.1016/j.jff.2020.104149 32837538 PMC7427583

[17] Yang HY Han L Lin YQ Li T Wei Y Zhao LH . Probiotic fermentation of herbal medicine: progress, challenges, and opportunities. Am J Chin Med. (2023) 51:1105–26. doi: 10.1142/S0192415X23500519 37357176

[18] Dong H Bu D Cheng Y Gao W Han F. Comprehensive advances on probiotic-fermented medicine and food homology. Fermentation. (2025) 11:682. doi: 10.3390/fermentation11120682

[19] Ali A Ahmad U Akhtar J Badruddeen Khan MM. Engineered nano scale formulation strategies to augment efficiency of nutraceuticals. J Funct Foods. (2019) 62:103554. doi: 10.1016/j.jff.2019.103554

[20] Ma J Wang J Wan Y Wang S Jiang C. Probiotic-fermented traditional Chinese herbal medicine, a promising approach to maintaining the intestinal microecology. J Ethnopharmacol. (2025) 337:118815. doi: 10.1016/j.jep.2024.118815 39270882

[21] Zhang Q Li YC Li JZ. Identification and enzyme production characteristics analysis of a Chinese herbal biotransformation probiotic. Heilongjiang Anim Sci Vete Med. (2017) 10:225–7, 98–99. doi: 10.13881/j.cnki.hljxmsy.2017.1837

[22] Guo F. Dong Y, Zhang T. Experimental study on herb residue gasification in anair-blown circulating fluidized bed gasifier. Ind Eng Chem Res. (2014) 54:13264–73. doi: 10.1021/ie5021238

[23] Wang J. Research on pretreatment and open fermentation of *Sophora flavescens* residues for L-lactic acid production. Univ Sci Technol Beijing. (2017).

[24] Zhang HG Teng J Li SX. Research progress of comprehensive utilization of chinese medicine residue for resources conservation. Guangzhou Chem Ind. (2013) 41:16–8.

[25] Deng HY Chen L Li WB. Preliminary study on the resource utilization of Chinese Medicine residue in the environment. Environ Dev. (2018) 6–7. doi: 10.16647/j.cnki.cn15-1369/X.2018.01.003.remain3

[26] Zhang ZD Bai HD Ke DS. Advances in modern research on fermentation of traditional chinese medicine. Asia-Pacific Tradit Med. (2024) 20:232–8.

[27] Min XB. Research on the function and efficiency mechanism of lactic acid bacteria. Ind Microbiol. (2023) 53:50–2. doi: 10.3969/j.issn.1001-6678.2023.03.017

[28] Zhou J Cheng J Liu L Luo J Peng X. *Lactobacillus acidophilus* (LA) fermenting astragalus polysaccharides (APS) improves calcium absorption and osteoporosis by altering gut microbiota. Foods. (2023) 12:275. doi: 10.3390/foods12020275 36673366 PMC9858548

[29] Zhang Y Shi C Wang C Lu Z Wang F Wang Y. PSXIV-42 Effect of a corn-soybean meal mixed feed fermented with Bacillus subtilis and Enterococcus faecium on intestinal morphrage, digestive function and flora of piglets. J Anim Sci. (2018) 96:42. doi: 10.1093/jas/sky404.094

[30] Bose S Kim H. Evaluation of *in vitro* anti-inflammatory activities and protective effect of fermented preparations of Rhizoma atractylodis macrocephalae on intestinal barrier function against lipopolysaccharide insult. Evid Based Complement Alternat Med. (2013) 2013:1–16. doi: 10.1155/2013/363076 23573125 PMC3612467

[31] Niu Y Wan XL Zhang LL Wang C He JT Bai KW . Effect of different doses of fermented Ginkgo biloba leaves on serum biochemistry, antioxidant capacity hepatic gene expression in broilers. Anim Feed Sci Technol. (2019) 248:132–40. doi: 10.1016/j.anifeedsci.2019.01.003

[32] Mao J Wang Y Duan T Yin N Dong C Ren X . Effect of fermented dandelion on productive performance, meat quality, immune function, and intestinal microbiota of broiler chickens. BMC Vet Res. (2023) 19:178. doi: 10.1186/s12917-023-03751-9 37773158 PMC10540353

[33] Anigboro AA Ajoh AI Avwioroko OJ Ehwarieme DA. Solid-state fermentation of cassava (Manihot esculenta) peels Ising *Rhizopus Oligosporus*: application of the fermented peels in yeast production and characterization of α-amylase enzyme produced in the process. Chemistry Africa. (2023) 6:1669–78. doi: 10.1007/s42250-022-00582-3

[34] Sun D Jin X Shi B Su J Tong M Yan S. Dietary Yucca schidigera extract improved growth performance and liver antioxidative function in broilers. Ital J Anim Sci. (2017) 16:677–84. doi: 10.1080/1828051X.2017.1302826

[35] Wang Q Wang L Li L Sun M Li P Yu Y . Effects of dietary supplementation of fermented Artemisia argyi on growth performance, slaughter performance, and meat quality in broilers. Poult Sci. (2024) 103:103545. doi: 10.1016/j.psj.2024.103545 38387294 PMC10899031

[36] Qu QS Li ZX Zhou Q. Research progress on fermented traditional Chinese medicine and its theoretical exploration of “fermentation compatibility”. Chin Tradit Herb Drugs. (2023) 54:2262–73. doi: 10.7501/j.issn.0253-2670.2023.07.027

[37] Jiao YH Gao TY Gong JG. Study on optimization of solid-state fermentation with mixed bacteria to improve nutritional value of seabuckthorn fruit residue. Feed Res. (2024) 24:75–9.

[38] Zhou MT Zhang YG Song ZY. Enhanced bioactivity of honeysuckle-Cassia seeds extracts through *Lactobacillus acidophilus* and *Bacillus subtilis* co-fermentation: Impact on alcoholic liver disease and gut microbiota. Food Chem. (2025) 486:144463. doi: 10.1016/j.foodchem.2025.145200 40339419

[39] Sheih IC Fang TJ Wu TK Chang CH Chen RY. Purification and properties of a novel phenolic antioxidant from Radix astragali fermented by *Aspergillus oryzae* M29. J Agric Food Chem. (2011) 59:6520–5. doi: 10.1021/jf2011547 21557623

[40] Liao YQ Kang YG Chang LL. Effects of bacterial enzyme coordinated fermentation of medicinal residue on growth performance,immune indexes and serum biochemical indexes of lambs. Chin Feed. (2025) 132–7. doi: 10.15906/j.cnki.cn11-2975/s.2024010021-01

[41] Liu FM Tan XD Yang YJ Duan YN. Production of protein feedstuff from notoginseng residues by solid-state fermentation. China Brewing. (2011) 2:67–70.

[42] Zhuang Y Xie XM Zhang LY. The origin development and i'ts advantage and potential of “the bi-directional solid fementation” for medicinal fungi. Chin edible mushrooms. (2006) 26:1–6.

[43] Li HX Zhou YK Fang PQ. Enhancement of antioxidant activity of chinese yam by fermentation with Cordyceps militaris. Food Sci. (2021) 42:51–6. doi: 10.7506/spkx1002-6630-20200419-245

[44] Liu ZY Qu QS Yang F. Study on changes of component content and antibacterial activity of mcoplasma in Bi-Directional fermentation pocess of Huaier and Radix Isatidis. Glob Tradit Chin Med. (2019) 12:1799–804.

[45] Zhuang Y Xie X. Primary studies of toxicity-reducing and efficacymaintaining action of fungal fermentative products in Tripterygium wilfordii by a novel bi-directional solidstate fungal fermentation. Zhongguo Zhong Yao Za Zhi. (2009) 34:2083–7. 19938552

[46] Zhang ZL Gao YG Zang P. Research progress on active components of fungi transforming Chinese materia medica. Chin Tradit Herb Drugs. (2019) 50:2736–42. doi: 10.7501/j.issn.0253-2670.2019.11.034

[47] Tu X Pan Y. The bi-directional fermentation technology, a new approach to attenuating the toxicity of toxic chinese materia medica. J Fungal Res. (2010) 8:52–6.

[48] Wang XB Feng X Liu Y. Research progress of multi-strains koji in soy sauce fermentation. Food Ferment Technol. (2016) 52:61–4. doi: 10.3969/j.issn.1674-506X.2016.03-014

[49] Jin SX Zhang GZ Lei LX. Effects of fermentation process of mixed bacteria on the nutrient components and polysaccharide content of supplementary SiJunZi decoction. Chin J Vet Med. (2015) 51:89–93.

[50] Jin SX Zhang GZ Lei LX. Study on the fermentation process parameters of mixed bacteria in modified Sijunzi Tang. Chin J Vet Med. (2015) 51:88–90.

[51] Qiao H Zhang X Shi H Song Y Bian C Guo A. Assessment of the physicochemical properties and bacterial composition of *Lactobacillus plantarum* and *Enterococcus* faecium-fermented Astragalus membranaceus using single molecule, real-time sequencing technology. Sci Rep. (2018) 8:11862. doi: 10.1038/s41598-018-30288-xpgvol 30089930 PMC6082834

[52] Li ZX. Preliminary study on the fermentation probiotics of residue fromJianweixiaoshi Tablets and the flora amelioration of the intestinaltract of spleen deficiency mice. Nanchang Univ. (2012).

[53] Zhao X Chen T Meng F Wang H Tian P Tang X . Therapeutic effect of herb residue fermentation supernatant on spleen-deficient mice. Mol Med Rep. (2018) 17:2764–70. doi: 10.3892/mmr.2017.8150 29207096

[54] Yuan MG Xiang R Peng XY. Research progress on production of functional feed by solid-state fermentation of traditional Chinese medicine residues. China Brewing. (2020) 39:17–20.

[55] Sun HQ. Sereening strains and optimizing conditions of *Astragalus* residue solid-state fermentation. Tianjin Univ Sci Technol. (2011).

[56] Tan XD Wang JJ Xu RF. Study on strain combination optimization for production of protein feed from notoginseng residues by mixed fermentation. China Brewing. (2012) 31:79–81.

[57] Liu YQ Li DW Li YF. The production technology research about using sophora japonica fermentation from liquid to solid production of livestock. Genomics Appl Biol. (2015) 35:787–91.

[58] Huang SP Gu J Yang XX. The effects of red ginseng dregs product of fermentation on serum biochemical index and organs on rats. Chin J Vet Med. (2017) 53:81–93.

[59] Tan XD Xu R Wang L. Study on producing protein feed from notoginseng residues in tray fermentor. Environ Sci Technol. (2015) 38:97–101.

[60] Tan XD DY Wang JJ. Study on fermentation process of different strains cultured in the substrate of notoginseng residues. J Food Sci Biotechnol. (2013) 32:882–6.

[61] Wang SJ Zhang LX Li PW. Antibacterial effects of perilla seed oil against *Escherichia Coli* and *Bacillus Subtilis*. Food Nutr China. (2017) 23:41–4.

[62] He F. Study on efficacy of the Chinese herb residues in Ancient Han Health Lotion through fermentation treatment in mice. Hunan Agric Univ. (2015).

[63] Liu X Guan B Hu Z Hu X Liu S Yang K . Combined traditional Chinese medicine and probiotics (TCMP) alleviates lipid accumulation and improves metabolism in high-fat diet mice via the microbiota-gut-liver axis. Food Res Int. (2025) 207:116064. doi: 10.1016/j.foodres.2025.116064 40086971

[64] Wang HR. The effect of Chinese medicine on the growth of probiotics and the design and optimization of Chinese medicineprobiotic composite microecological preparation. Jilin University. (2020).

[65] Wu JP WuY Zhang J. To optimize the preparation process of massa medicata fermentata by probiotic fermentation based on response surface method. Agric Prod Process. (2025) 31–43.

[66] Han X. Digestive and gut metabolic characteristics of hawthorn procyanidins and its mechanism of relieving lipid metabolism disorder. Hebei Agric Univ. (2022).

[67] Guo Xin. The alleviation effect of three kinds of traditional Chinese medicine compound and their fermented decoction on dysbacteriotic diarrhea mice induced by antibiotic. Shanxi Agric Univ. (2021).

[68] Jiao QF. Study of microbial strains screeningfor transformation and utilizationof Chinese herbal medicine. Zhejiang Norm Univ. (2010).

[69] Guo JJ Xia N Zhuang X. The effect of solid-state fermentation of endophytic bacterium Ilyonectoria cyclosaminicola on the functional components of Epimedium herb residue. Lishizhen Med Mater Med Res. (2023) 34:428–31.

[70] Wang Y Liu CX Wang XY. Study on the variation of rosmarinic acid and other active ingredients in oregano residue feed fermentation. Chinese Feed. (2024) 123–130. doi: 10.15906/j.cnki.cn11-2975/s.2023070009-07

[71] Yan L Ma YX Li MQ. Metabolomics of *Lactiplantibacillus plantarum* fermentation on ginseng residues. Fujian J Agric Sci. (2025) 40:18–28. doi: 10.19303/j.issn.1008-0384.2025.01.003

[72] Moon K Cha J. Enhancement of antioxidant and antibacterial activities of salvia miltiorrhiza roots fermented with *Aspergillus oryzae*. Foods. (2020) 9:34. doi: 10.3390/foods9010034 31906298 PMC7023044

[73] He LY Lin ZC Lu JD. Detoxification and sustained effects of Tripterygium wilfordii based on Ganoderma lucidum bi-directional solid fermentation. J Beijing Univ Chem Technol. (2021) 48:48–56.

[74] Hou ZF Lian YH He LB. Chemical constituents changes in Tripterygium wilfordii after ganoderma bidirectional solid fermentation. Chinese Tradit Herb Drugs. (2012) 234–7.

[75] Jung J Jang HJ Eom SJ Choi NS Lee NK Paik HD. Fermentation of red ginseng extract by the probiotic Lactobacillus plantarum KCCM 11613P: ginsenoside conversion and antioxidant effects. J Ginseng Res. (2019) 43:20–6. doi: 10.1016/j.jgr.2017.07.004 30662290 PMC6323145

[76] Xia Q Zhang XX Xu KX. Review on toxicity of toxic traditional Chinese medicine recorded in Chinese pharmacopoeia (2015 version). Glob Tradit Chin Med. (2017) 10:377–84.

[77] Wang DS Liu GL Yang G. Research progress on technology related to improving dairy feed palatability. Feed Feed. (2016) 14–8. doi: 10.19305/j.cnki.11-3009/s.2016.07.004

[78] Xue R Gong JL Zhang W. Research progress and discussion on processing of toxic traditional Chinese medicine decoction pieces. World Chin Med. (2022) 17:1193–201.

[79] Jiang N Wei W Xu XY. Construction of solid fermentation of traditionalchinese medicine Radix Aconiti. J Sichuan Unv. (2013) 50:1105–9.

[80] Peng W Sun Y Pan Y. Effects of different fermentation days on acute toxicity and analgesic, anti-Inflammatory activity of shuanqian junzhi. J Food Sci Biotechnol. (2013) 32:541–5.

[81] Mohd Redzwan S Abd Mutalib MS Wang JS Ahmad Z Kang MS Abdul Rahman N' . Effect of supplementation of fermented milk drink containing probiotic Lactobacillus casei Shirota on the concentrations of aflatoxin biomarkers among employees of Universiti Putra Malaysia: a randomised, double-blind, cross-over, placebo-controlled study. Br J Nutr. (2016) 115:39–54. doi: 10.1017/S0007114515004109 26490018

[82] Li L Wang L Fan W Jiang Y Zhang C Li J . The application of fermentation technology in traditional Chinese medicine: a review. Am J Chin Med. (2020) 48:899–921. doi: 10.1142/S0192415X20500433 32431179

[83] Tang N Xu X Guo Z Meng X Qian G Li H. Preparation of safflower fermentation solution and study on its biological activity. Front Microbiol. (2024) 15:1472992. doi: 10.3389/fmicb.2024.1472992 39539711 PMC11557484

[84] Lee YR Cha YJ Jeong S Yun SK Nho Y Kang S . A novel sophorolipids extraction method by yeast fermentation process for enhanced skin efficacy. Skin Res Technol. (2023) 29:e13518. doi: 10.1111/srt.13518 38009026 PMC10643984

[85] Zhang WY. Study on the lipid-lowering effect of traditional Chinese medicine and Ganoderma lucidum bidirectional deep fermentation system. Beijing University of Chinese Medicine. (2017).

[86] Zang XL Ma C Hu YL. Research progress on the lipid-lowering effect and application of fermented foods with traditional Chinese medicine. Nutr Health Care. (2024) 96–8. doi: 10.12325/j.issn.1672-

[87] Alam S Liaqat I Al-Arifa N Zia T Munawar M Muzamil A. Obesity theranostics using nanoemulsions of probiotics and local herbs. Saudi J Biol Sci. (2023) 30:103790. doi: 10.1016/j.sjbs.2023.103790 37680978 PMC10480777

[88] Chen JY. Screening of lipid-lowering probiotics and evaluation of their combined effects with fermented Chinese herbal medicine. Kunming University of Science and Technology. (2024).

[89] Yang HL. Study on the lipid-lowering effect and mechanism of polysaccharide from Smiax glabra Roxb on caenorhabditis elegans. Guangzhou University of Chinese Medicine. (2020).

[90] Zhou P Song C Chi SH Feng J. Extraction technology of traditional Chinese medicine. J Tradit Chin Vet Med. (2022) 41:37–43.

[91] Park YJ Kim MS Kim HR Kim JM Hwang JK Yang SH . Ethanol extract of alismatis rhizome inhibits adipocyte differentiation of OP9 cells. Evid Based Complement Alternat Med. (2014) 2014:415097. doi: 10.1155/2014/415097 25013444 PMC4070469

[92] Shi H Zheng Y Zhao J Li Y Jia H Hou X . Zexie decoction reduce glucose-dependent lipid accumulation and oxidative stress in Caenorhabditis elegans. Phytomedicine. (2023) 120:155036. doi: 10.1016/j.phymed.2023.155036 37643530

[93] Mei L Tang Y Li M Yang P Liu Z Yuan J . Co-administration of cholesterol-lowering probiotics and anthraquinone from cassia obtusifolia L. ameliorate non-alcoholic fatty liver. PloS ONE. (2015) 10:e0138078. doi: 10.1371/journal.pone.0138078 26375281 PMC4573521

[94] Wang M Sun H Wang X Zhang X Huang Y Cui R . Tangerine peel-based herbal formula ameliorates metabolic syndrome via gut microbiota-mediated bile acid remodeling and TGR5 activation. Am J Chin Med. (2025) 53:2541–59. doi: 10.1142/S0192415X25500946 41263038

[95] Hu J Lin S Zheng B Cheung PCK. Short-chain fatty acids in control of energy metabolism. Crit Rev Food Sci Nutr. (2018) 58:1243–9. doi: 10.1080/10408398.2016.1245650 27786539

[96] Martin-Gallausiaux C Marinelli L Blottière HM Larraufie P Lapaque N. SCFA: mechanisms and functional importance in the gut. Proc Nutr Soc. (2021) 80:37–49. doi: 10.1017/S0029665120006916 32238208

[97] Ratajczak W Rył A Mizerski A Walczakiewicz K Sipak O Laszczyńska M. Immunomodulatory potential of gut microbiome-derived short-chain fatty acids (SCFAs). Proc Nutr Soc. (2019) 66:1–12. doi: 10.18388/abp.2018_2648 30831575

[98] Zhang HY Tian JX Lian FM Li M Liu WK Zhen Z . Therapeutic mechanisms of traditional Chinese medicine to improve metabolic diseases via the gut microbiota. Biomed Pharmacother. (2021) 133:110857. doi: 10.1016/j.biopha.2020.110857 33197760

[99] WWahabi S Rtibi K Atouani A Sebai H. Anti-obesity actions of two separated aqueous extracts from arbutus (arbutus unedo) and hawthorn (crataegus monogyna) fruits against high-fat diet in rats via potent antioxidant target. Dose Response. (2023) 21:15593258231179904. doi: 10.1177/15593258231179904 37275393 PMC10236257

[100] Wang Y Fei Y Liu L Xiao Y Pang Y Kang J . Polygonatum odoratum polysaccharides modulate gut microbiota and mitigate experimentallyninduced obesity in rats. Int J Mol Sci. (2018) 19:3587. doi: 10.3390/ijms19113587 30428630 PMC6274832

[101] Ou X Chen J Li B Yang Y Liu X Xu Z . Multiomics reveals the ameliorating effect and underlying mechanism of aqueous extracts of polygonatum sibiricum rhizome on obesity and liver fat accumulation in high-fat diet-fed mice. Phytomedicine. (2024) 132:155843. doi: 10.1016/j.phymed.2024.155843 38971026

[102] Feng Q Lin J Niu Z Wu T Shen Q Hou D . A comparative analysis between whole chinese yam and peeled chinese yam: their hypolipidemic effects via modulation of gut microbiome in high-fat diet-fed mice. Nutrients. (2024) 16:977. doi: 10.3390/nu16070977 38613011 PMC11013417

[103] Zheng H Ji H Fan K Xu H Huang Y Zheng Y . Targeting gut microbiota and host metabolism with dendrobium officinale dietary fiber to prevent obesity and improve glucose homeostasis in diet-induced obese mice. Mol Nutr Food Res. (2022) 66:e2100772. doi: 10.1002/mnfr.202100772 35225418

[104] Huang S Zou Y Tang H Zhuang J Ye Z Wei T . Cordyceps militaris polysaccharides modulate gut microbiota and improve metabolic disorders in mice with diet-induced obesity. J Sci Food Agric. (2023) 103:1885–94. doi: 10.1002/jsfa.12409 36571152

[105] Qin X Fang Z Zhang J Zhao W Zheng N Wang X. Regulatory effect of Ganoderma lucidum and its active components on gut flora in diseases. Front Microbiol. (2024) 15:1362479. doi: 10.3389/fmicb.2024.1362479 38572237 PMC10990249

[106] Lan Y Sun Q Ma Z Peng J Zhang M Wang C . Seabuckthorn polysaccharide ameliorates high-fat diet-induced obesity by gut microbiota-SCFAs-liver axis. Food Funct. (2022) 13:2925–37. doi: 10.1039/D1FO03147C 35191457

[107] Guo C Han L Li M Yu L. Seabuckthorn (Hippophaë rhamnoides) freeze-dried powder protects against high-fat diet-induced obesity, lipid metabolism disorders by modulating the gut microbiota of mice. Nutrients. (2020) 12:265. doi: 10.3390/nu12010265 31968607 PMC7020008

[108] Zhao JD Yu CJ Cheng RD Li JJ Fang ZH. Research progress in the regulation of glucose metabolism with herbs based on gut microbiota-bile acid pathway. West J Tradit Chin Med. (2025) 38:103–7.

[109] Huo XK Liu J Yu ZL. Alisma orientale extract exerts the reversing cholestasis effect by activation of farnesoid X receptor. Phytomedicine. (2018) 42:34–42. doi: 10.1016/j.phymed.2018.03.017 29655695

[110] Yan P Wei Y Wang M. Network pharmacology combined with metabolomics and lipidomics to reveal the hypolipidemic mechanism of Alismatis rhizoma in hyperlipidemic mice. Food Funct. (2022) 13:4714–33. doi: 10.1039/D1FO04386B 35383784

[111] Li M Zhao Y Wang Y Geng R Fang J Kang SG . Eugenol, a major component of clove oil, attenuates adiposity, and modulates gut microbiota in high-fat diet-fed mice. Mol Nutr Food Res. (2022) 66:e2200387. doi: 10.1002/mnfr.202200387 36029106

[112] Liao C Tang CL Qiu W. Electroacupuncture activates PKA / CREB signaling pathway to promote browning of white fat in obese rats. Acupunct Res. (2025) 50:553–66. doi: 10.13702/j.1000-0607.20240755 40390613

[113] Yang DM Zhang XY Ji ZX. Effect of aqueous extract of *Poria cocos* and *Coix seed* on obese mice. Food Res Dev. (2025) 46:12–21.

[114] Zhou Y Teng JQ Tian LL. Research progress on the intervention mechanism of Danshen and its effective chemical components on obesity. J Chin Med Mater. (2023) 45:2890–4.

[115] Meikle PJ Summers SA. Sphingolipids and phospholipids in insulin resistance and related metabolic disorders. Nat Rev Endocrinol. (2017) 13:79–91. doi: 10.1038/nrendo.2016.169 27767036

[116] Shi NN. Effect of fermented barley extract on lipometabolism in 3T3-L1 preadipocytes and its mechanism. Jiangsu University. (2016).

[117] Jin L. A research into Gegen Qinlian decoction fermentation broth's functions of declining T2DM Rats' sugar and fat and its mechanism. Hubei University of Chinese Medicine. (2016).

[118] JJoung H Kim B Park H Lee K Kim HH Sim HC . Fermented moringa oleifera decreases hepatic adiposity and ameliorates glucose intolerance in high-fat diet-induced obese mice. J Med Food. (2017) 20:439–47. doi: 10.1089/jmf.2016.3860 28504910

[119] Lee HS Lim WC Lee SJ Lee SH Lee JH Cho HY. Antiobesity effect of garlic extract fermented by Lactobacillus plantarum BL2 in diet-induced obese mice. J Med Food. (2016) 19:823–9. doi: 10.1089/jmf.2016.3674 27627701

[120] Mao K Liu H Cao X Yang M Wang X Yang X . Hawthorn or semen cassiae-alleviated high-fat diet-induced hepatic steatosis in rats via the reduction of endoplasmic reticulum stress. Food Funct. (2022) 13:12170–81. doi: 10.1039/D2FO02487J 36326424

[121] Wang Y Yang J Wang W Sanidad KZ Cinelli MA Wan D . Soluble epoxide hydrolase is an endogenous regulator of obesity-induced intestinal barrier dysfunction and bacterial translocation. Proc Natl Acad Sci USA. (2020) 117:8431–6. doi: 10.1073/pnas.1916189117 32220957 PMC7165420

[122] Chen H Yang H Deng J Fan D. Ginsenoside Rk3 ameliorates obesity-induced colitis by regulating of intestinal flora and the TLR4/NF-κB signaling pathway in C57BL/6 mice. J Agric Food Chem. (2021) 69:3082–93. doi: 10.1021/acs.jafc.0c07805 33621094

[123] Xu Y Wang N Tan HY Li S Zhang C Zhang Z . Panax notoginseng saponins modulate the gut microbiota to promote thermogenesis and beige adipocyte reconstruction via leptin-mediated AMPKα/STAT3 signaling in diet-induced obesity. Theranostics. (2020) 10:11302–23. doi: 10.7150/thno.47746 33042284 PMC7532683

[124] An SJ. The intervention effect of Cornus officinalis and loganin on obese mice and its impact on gut microbiota. Shanxi Normal University. (2024).

[125] Wu L Yan Q Chen F Cao C Wang S. Bupleuri radix extract ameliorates impaired lipid metabolism in high-fat diet-induced obese mice via gut microbia-mediated regulation of FGF21 signaling pathway. Biomed Pharmacother. (2021) 135:111187. doi: 10.1016/j.biopha.2020.111187 33556916

[126] Han K Bose S Wang JH Lim SK Chin YW Kim YM . *In vivo* therapeutic effect of combination treatment with metformin and Scutellaria baicalensis on maintaining bile acid homeostasis. PLoS ONE. (2017) 12:e0182467. doi: 10.1371/journal.pone.0182467 28877164 PMC5587228

[127] Hasani-Ranjbar S Hoseini Tavassol Z Malmir H Ejtahed HS Tajabadi Ebrahimi M Larijani B. Investigation of the probiotic supplement's effect on obese adults demonstrated a reduction in fasting insulin levels: a double-blind randomized clinical trial. J Diabetes Metab Disord. (2024) 23:1141–9. doi: 10.1007/s40200-024-01400-y 38932862 PMC11196508

[128] Fan Y Liu Y Shao C Jiang C Wu L Xiao J . Gut microbiota-targeted therapeutics for metabolic disorders: mechanistic insights into the synergy of probiotic-fermented herbal bioactives. Int J Mol Sci. (2025) 7:26. doi: 10.3390/ijms26125486 40564947 PMC12193472

[129] Qureshi R Picon-Ruiz M Aurrekoetxea-Rodriguez I Nunes de Paiva V D'Amico M Yoon H . The major pre- and postmenopausal estrogens play opposing roles in obesity-driven mammary inflammation and breast cancer development. Cell Metab. (2020) 31:1154–72.e9. doi: 10.1016/j.cmet.2020.05.008 32492394

[130] Lyon J. 3rd, Connell M, Chandrasekaran K, Srivastava S. Effect of synbiotics on weight loss and metabolic health in adults with overweight and obesity: A randomized controlled trial. Obesity. (2023) 31:2009–20. doi: 10.1002/oby.23801 37424169

[131] Teo YQJ Chong B Soong RY Yong CL Chew NW Chew HSJ. Effects of probiotics, prebiotics and synbiotics on anthropometric, cardiometabolic and inflammatory markers: An umbrella review of meta-analyses. Clin Nutr. (2024) 43:1563–83. doi: 10.1016/j.clnu.2024.05.019 38754308

[132] Wang ZB Xin SS Ding LN Ding WY Hou YL Liu CQ . The potential role of probiotics in controlling overweight/obesity and associated metabolic parameters in adults: a systematic review and meta-analysis. Evid Based Complement Alternat Med. (2019) 2019:3862971. doi: 10.1155/2019/3862971 31118956 PMC6500612

[133] Huang M Cople-Rodrigues CDS Waitzberg DL Rocha I Curioni CC. Changes in the gut microbiota after the use of herbal medicines in overweight and obese individuals: a systematic review. Nutrients. (2023) 5:15. doi: 10.3390/nu15092203 37432344 PMC10181072

[134] Guedes MR Pontes K Barreto Silva MI Neves MF Klein M. Randomized controlled trials reporting the effects of probiotics in individuals with overweight and obesity: a critical review of the interventions and body adiposity parameters. Clin Nutr. (2023) 42:835–47. doi: 10.1016/j.clnu.2023.03.017 37084470

[135] Sun N Zhao Y Zhang A He Y. Gut microbiota and osteoarthritis: epidemiology, mechanistic analysis, and new horizons for pharmacological interventions. Front Cell Infect Microbiol. (2025) 15. doi: 10.3389/fcimb.2025.1605860 40740348 PMC12307352

[136] Gui R. Effects of rehmannia glutinosa oligosaccharides on intestinal inflammation and barrier damage induced by LPS in mice. Henan Agricultural University. (2024).

[137] Wang L Wu Y Zhuang L Chen X Min H Song S . Puerarin prevents high-fat diet-induced obesity by enriching Akkermansia muciniphila in the gut microbiota of mice. PLoS ONE. (2019) 14:e0218490. doi: 10.1371/journal.pone.0218490 31233515 PMC6590871

[138] Dong PT Li XY Zhang Q. Zhen Y, Zhigang A, Jianjun W. Research progress on the mechanism of flavonoids of traditional Chinese medicine in improving diabetes nephropathy. Chin Tradit Pat Med. (2024) 47:3337–45.

[139] Lee JY Park HM Kang CH. Antioxidant effect via bioconversion of isoflavonoid in astragalus membranaceus fermented by Lactiplantibacillus plantarum MG5276 *in vitro* and *in vivo*. Fermentation. (2022) 8:34. doi: 10.3390/fermentation8010034

[140] Li Y Liu H Qi H Tang W Zhang C Liu Z . Probiotic fermentation of Ganoderma lucidum fruiting body extracts promoted its immunostimulatory activity in mice with dexamethasone-induced immunosuppression. Biomed pharmacother. (2021) 141:111909. doi: 10.1016/j.biopha.2021.111909 34328088

[141] Yang M Tao L Zhao CC Wang ZL Yu ZJ Zhou W . Antifatigue effect of panax notoginseng leaves fermented with microorganisms: *in-vitro* and *in-vivo* evaluation. Front Nutr. (2022) 9:824525. doi: 10.3389/fnut.2022.824525 35273989 PMC8904179

[142] Song YQ Chen YW. Study on the chemical components of transforming *Puerariae* radix with *Monascus*. Nat Prod Res Dev. (2013) 25:1525–8.

[143] Gao J Dong WB Wang Y. Optimization of fermentation conditions for conversion of conjagated anthraquionone into free anthraquionone in Dahuang. Introd Tradit Chin Med. (2017) 23:59–61.

[144] Yan Y Du C Li Z Zhang M Li J Jia J . Comparing the antidiabetic effects and chemical profiles of raw and fermented Chinese Ge-Gen-Qin-Lian decoction by integrating untargeted metabolomics and targeted analysis. Chin Med. (2018) 13:54. doi: 10.1186/s13020-018-0208-7 30386417 PMC6204051

[145] Tang T Wang Q Zhou WQ. Research analysis and application of solid-state fermentation technology. Ind Microbiol. (2020) 50:55–9.

[146] Han SW. Research on preparation process and feeding effectiveness in chicken of fermented Astragalus powder. Chinese Academy of Agricultural Sciences Thesis. (2024).

[147] Su GL Zhang JY Zhang K. Study on improving active ingredients of astragalus root,stem and leaf by probiotic fermentation. Chin Anim Husb Vet Med. (2017) 44:1877–83.

[148] Yan XT Zhang W Zhang Y Zhang Z Chen D Wang W . *In vitro* anti-obesity effect of Shenheling Extract (SHLE) fermented with Lactobacillus fermentum grx08. Foods. (2022) 11:1221. doi: 10.3390/foods11091221 35563944 PMC9104015

[149] Li BZ Liu Q Fan JH. Optimization of conditions for increasing the contents of astragalus polysaccharide by microbial fermentation. Chin J Vet Drug. (2022) 56:68–74.

[150] An G Chen B Zhang C Zhong H Wang J Zhang Z . Extraction process, structural characterization, and digestive properties of type 3 resistant starch from Angelica Dahurica, and its impact on gut health in obese mice. Int J Biol Macromol. (2025) 319:145598. doi: 10.1016/j.ijbiomac.2025.145598 40581011

[151] Kim H Suh HJ Kang CM Lee KH Hwang JH Yu KW. Immunological activity of ginseng is enhanced by solid-state culture with Ganoderma lucidum mycelium. J Med Food. (2014) 17:150–60. doi: 10.1089/jmf.2013.3063 24456366

[152] Xin YH Liang B Wang YX. Optimization of the Ganoderma lucidum-Ginkgobiloba bi-directional liquid fermentation condition and antioxidation properties of itsproducts. Mycosystema. (2017) 36:1427–35.

[153] Sun WP. The study of bi-directional solid state fermentation for transforming inonotus obliquus with monascus and the pharmacodynamics of mycoplasm. Yanbian University. (2015).

[154] Zhao YX Ni AX Zhou LH Xie J. Optimization of bidirectional fermentation conditions of Monascus sp. and Dendrobium nobile Genomics and Applied Biology. (2021) 40:686–94.

[155] Zou G Nielsen JB Wei Y. Harnessing synthetic biology for mushroom farming. Trends Biotechnol. (2023) 41:480–3. doi: 10.1016/j.tibtech.2022.10.001 36307231

[156] Wang X Li Y Yu P Zhang L Wang Y Yang X . Eurotium Cristatum fermented instant dark tea prevents obesity and promotes adipose thermogenesis via modulating the gut microbiota. Food Res Int. (2025) 219:116969. doi: 10.1016/j.foodres.2025.116969 40922197

[157] Zhang R Li Q Gu Y Liao W. Harnessing the power of fermented tea to improve gut microbiota and combat obesity epidemic. Biology. (2024) 13:779. doi: 10.3390/biology13100779 39452088 PMC11504357

[158] Jiang ZW Huang HQ Wu HT. The therapeutic mechanisms of gut microbiota regulated by traditional chinese medicines for obesity epidemic. Pharm Inf. (2021) 10:204–8. doi: 10.12677/PI.2021.104026

